# Challenges and Outcomes in the Management of Periprosthetic Humeral Fractures: A Retrospective Study and Review of Current Approaches

**DOI:** 10.7759/cureus.62534

**Published:** 2024-06-17

**Authors:** Anass Abaydi, Mohamed Kadi, Jihad Radi, Kamal Lahrach, Fawzi Boutayb

**Affiliations:** 1 Orthodontics Department, University Hospital Center Hassan II, Fes, MAR; 2 Traumatology and Orthopedic Surgery Department, University Hospital Center Hassan II, Fes, MAR

**Keywords:** lcp, prosthetic replacement, conservative and surgical treatment, shoulder arthroplasties, periprosthetic humeral fractures

## Abstract

Introduction: Periprosthetic humeral fractures are a rare and increasing entity due to the rising number of shoulder arthroplasties. These fractures pose a significant challenge for surgeons, with incidence rates ranging from 1.2% to 19.4%. They can occur intraoperatively or as late complications, often influenced by trauma, prosthetic wear, or loosening.

Patients and methods: A retrospective study was conducted on all patients admitted with periprosthetic humeral fractures over a four-year period (2018-2022). Inclusion criteria were postoperative periprosthetic humeral fractures with a minimum follow-up of six months. Exclusion criteria included intraoperative fractures, fractures of the glenoid or coracoid process, and cases with follow-up of less than six months or incomplete data.

Results: The study included six patients with an average age of 83.1 years, predominantly female (four females and two males). All fractures occurred postoperatively: four on reverse shoulder prostheses, one on an anatomical prosthesis, and one on a hemiarthroplasty. The mechanism was low-energy trauma, with fractures occurring an average of 96 months post-initial surgery. Fractures were classified using the Campbell system: three in region 4, two in region 3, and one in region 2. Radiographs showed four cemented and two uncemented stems. Three patients underwent surgical treatment with either prosthetic replacement using a long stem and fracture cerclage or locking compression plate (LCP). The remaining three patients were treated conservatively with a Sarmiento brace due to advanced age, bone fragility, low functional demand, and comorbidities. Radial nerve palsy was a complication in two patients post-trauma, with one recovering fully and the other not recovering before death due to associated complications. All fractures consolidated within an average of seven months (range: 5-8 months). Functional recovery was satisfactory with a median Constant-Murley Shoulder Score of 69 in surgically treated patients, with range of motion between 100 and 140 degrees. Only two conservatively treated patients achieved fracture consolidation, and functional recovery was inadequate.

Discussion: Managing periprosthetic humeral fractures remains challenging. Treatment goals include fracture healing, maintaining prosthetic stem stability, preserving glenohumeral motion, and restoring shoulder function. Despite various classification systems, the literature shows limited and variable data on incidence and treatment outcomes. Conservative treatment may be considered for stable implants and acceptable alignment, but surgical intervention is often necessary for displaced fractures or implant loosening.

Conclusion: The management of periprosthetic humeral fractures requires a tailored, multidisciplinary approach to optimize outcomes and improve patient quality of life. With the increasing incidence of these fractures due to the growing use of shoulder arthroplasty, ongoing research and development of new techniques and therapeutic strategies are essential to address this clinical challenge effectively.

## Introduction

The incidence of periprosthetic fractures of the humerus ranges between 1.2% and 19.4%, making them a rare but increasingly common challenge for surgeons due to the growing number of shoulder prostheses being implanted [1-3].

Intraoperative fractures are rare and typically result from technical errors, while late postoperative fractures can be associated with trauma, wear, or prosthetic loosening [4]. Various classification systems have been developed to guide treatment. According to Campbell et al. [5], fractures can be categorized by their location into four regions.

The treatment of periprosthetic humeral fractures depends on the timing of the fracture, its location relative to the humeral stem, and the stability of the implant [6]. The primary goal is to restore function to the limb and joint by ensuring bone healing and implant stability. Therapeutic options include conservative treatment or surgical intervention, which can be further divided into isolated osteosynthesis or prosthetic replacement with or without osteosynthesis. The therapeutic decision is guided by whether the implant is cemented or not, the quality of the bone stock, and the general condition of the patient, including comorbidities and functional expectations.

Our study aims to present our institution's experience in managing these fractures. The primary goal is to assess functional recovery, with a secondary focus on evaluating fracture healing resulting from the treatments administered.

## Materials and methods

To assess the treatment and results of periprosthetic humeral fractures in patients admitted to our facility during a four-year period, from 2018 to 2022, a retrospective analysis was carried out. To guide future clinical practice and enhance patient care, this study sought to collect thorough data on the prevalence, therapeutic modalities, and clinical outcomes of these fractures.

The inclusion criteria for this study were meticulously defined to ensure the relevance and reliability of the findings. All patients who experienced periprosthetic humeral fractures postoperatively were included, provided they had a minimum follow-up duration of six months. This criterion was crucial to allow adequate time for monitoring the healing process, assessing the effectiveness of the treatment, and evaluating long-term outcomes such as functional recovery and complication rates.

Conversely, specific exclusion criteria were applied to refine the study population and maintain focus on the target group. Patients who sustained intraoperative periprosthetic humeral fractures were excluded. Additionally, fractures involving the glenoid or coracoid process were excluded due to their distinct anatomical and clinical considerations. Cases with a follow-up period of less than six months were also excluded to avoid incomplete data. Instances of insufficient data, such as incomplete medical records or missing follow-up information, were similarly excluded to ensure the robustness and accuracy of the data analyzed.

A comprehensive examination of the patient's medical records, surgical reports, and follow-up visit notes was done to gather data. Patient demographics, fracture classification, specifics of the surgical or conservative treatment given, and postoperative care guidelines were among the important data that were noted. All complications, including hardware failure, non-union, and infection, were carefully recorded. Clinical parameters, such as pain thresholds and range of motion, along with radiographic proof of implant stability and fracture healing, were used to evaluate the results.

## Results

This retrospective study covered a period of four years. Our study group comprised six patients with an average age of 83.1 years, predominantly female (four females and two males). All fractures occurred after the initial surgery, including four fractures on reverse shoulder prostheses, one fracture on an anatomical prosthesis, and one fracture on hemiarthroplasty. The fractures in our patients resulted from low-energy mechanisms, with the average time from the initial surgery to the occurrence of the fracture being 96 months (range: 2-200 months).

**Figure 1:**
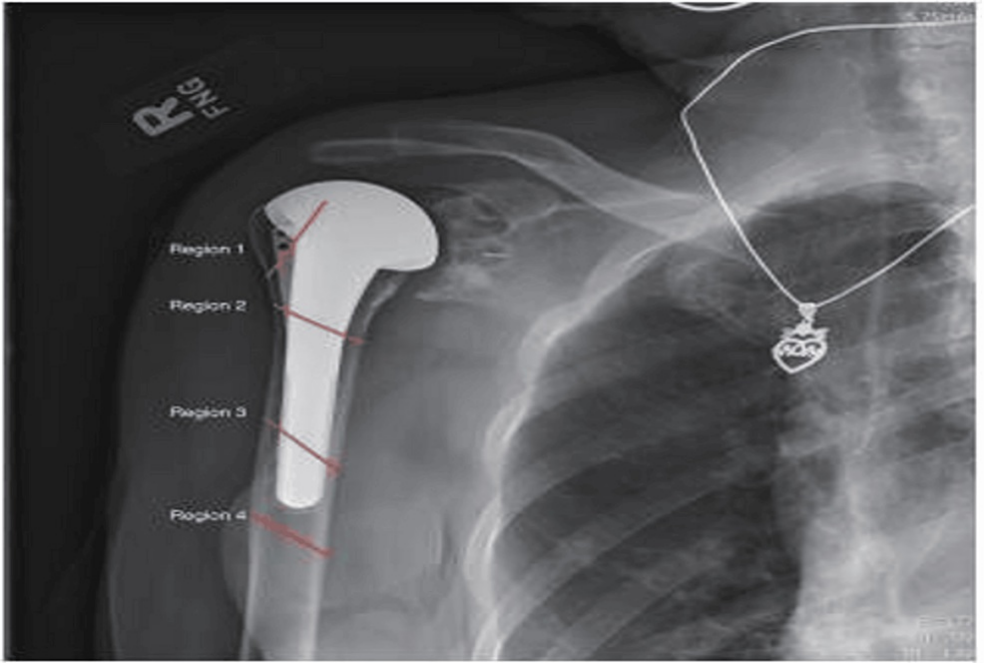
Campbell's classification: region 1: tuberosity fractures, region 2: proximal metaphyseal fractures of the humerus, region 3: proximal diaphyseal fractures of the humerus, and region 4: fracture of the mid or distal third of the humeral diaphysis.

**Figure 2:**
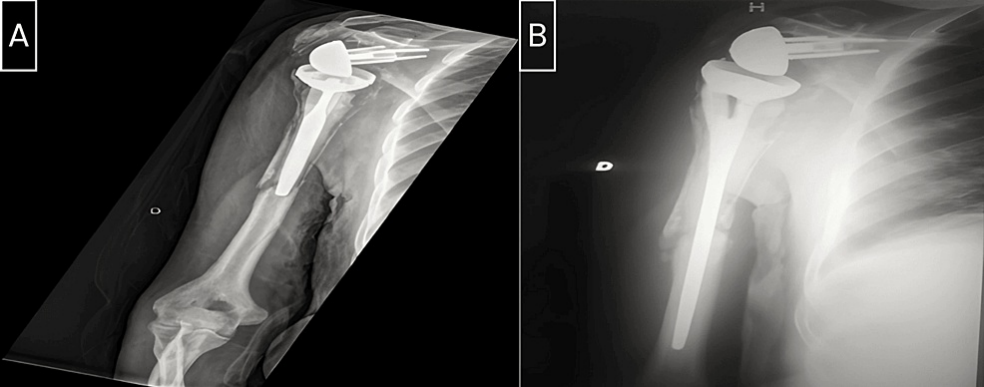
A: Frontal radiograph of the right shoulder shows a periprosthetic fracture of region 3. B: Frontal radiograph of the right shoulder shows the evolution after eight months post-revision.

**Figure 3:**
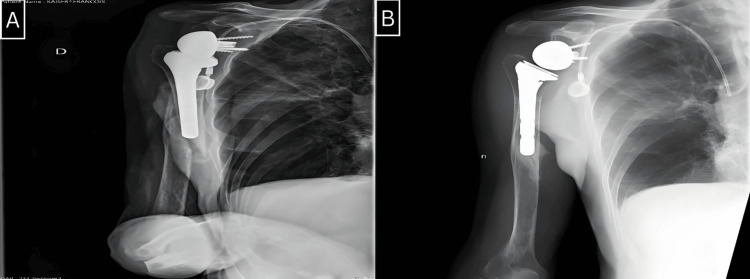
A: Frontal radiograph of the right shoulder shows a periprosthetic fracture of region 3. B: Frontal radiograph of the right shoulder shows the evolution after five months of orthopedic treatment.

**Figure 4:**
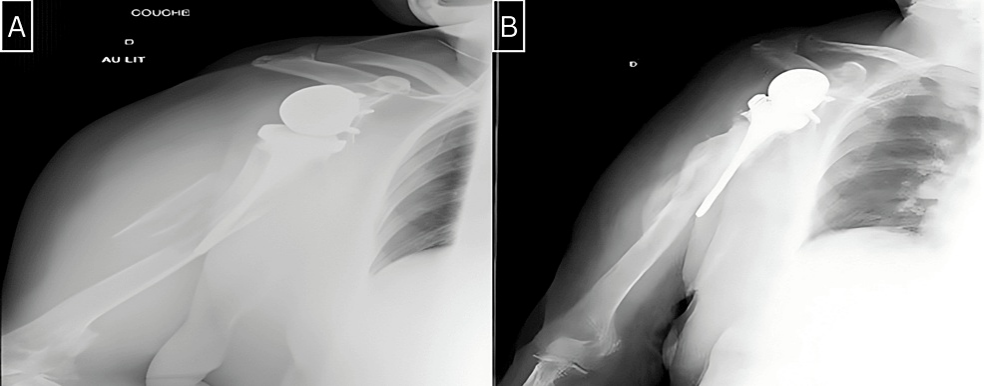
A: Frontal radiograph of the right shoulder shows a periprosthetic fracture of region 3. B: Frontal radiograph of the right shoulder shows the evolution after six months post-revision.

**Figure 5:**
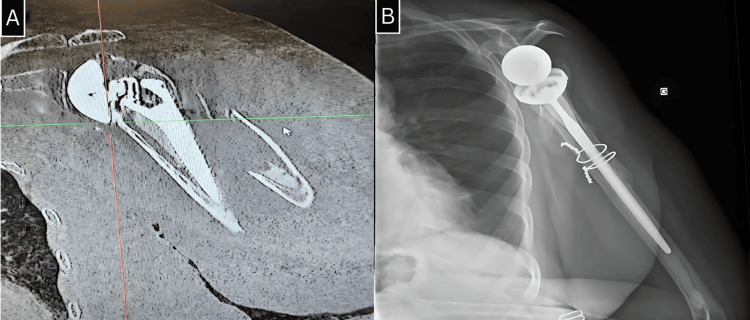
A: Frontal section of the CT scan image of a periprosthetic fracture of region 3 in the left shoulder. B: Frontal radiograph of the left shoulder shows the evolution after six months post-revision. CT: computed tomography

We utilized Campbell's classification to categorize the fractures in our series. There were three fractures in region 4, two in region 3, and one in region 2 (Figure 1). Radiographic analysis revealed that four stems were cemented, and two were uncemented (Figures 2-5).

Three patients in our study underwent surgical treatment, either through prosthetic replacement using a long stem with cerclage of the fracture site or through osteosynthesis with a locking compression plate (LCP). The remaining three patients were treated conservatively with a Sarmiento brace. This choice was primarily motivated by advanced age, bone fragility, low functional demand, and associated comorbidities that could contraindicate surgical treatment in these patients.

Radial nerve palsy was the primary complication in our series, affecting two patients after trauma. The first patient recovered normal function within three months, while the second patient showed no improvement and eventually passed away due to associated complications. There were no cases of postoperative nerve palsy or prosthetic infections in our series.

Fractures typically healed within an average of seven months (range: 5-8 months). Patients who had surgery showed good functional recovery, with an average shoulder elevation of 100 to 140 degrees. Among those treated without surgery, only two patients experienced complete healing of the fracture, but their functional recovery was inadequate. The characteristics of the patients included in this study are shown in Table 1.

**Table 1:** Characteristics of the patients included in the series. RTSA: reverse total shoulder arthroplasty, M: months, RP: radial paralysis, CKD: chronic kidney disease

Patient	Age	Sex	Risk factor	Indication	Arthroplasty	Mechanism	Type of fracture	Occurrence of fracture in months	Complication	Treatment	Consolidation in months	Functional recovery
1	76	Male	Osteoporosis, osteopenia, gastric carcinoma, hepatic cirrhosis	Glenohumeral osteoarthritis	Cementless RTSA	Fall	Region 4	2 M	-	Immobilization by Sarmiento cast	5 M	Beginning of functional recovery, currently undergoing rehabilitation
2	91	Female	Osteoporosis, cognitive impairment, CKD	Glenohumeral osteoarthritis	Cemented RTSA	Fall	Region 4	6 M	RP	Immobilization by Sarmiento cast	Highly displaced fracture	No recovery on the deficit after 6 months
3	80	Male	Osteoporosis	Glenohumeral osteoarthritis	Revision RTSA cement	Fall	Region 3	70 M	RP	RTSA surgery with long stem	8 M	No paralysis, mobility of elevation at 12°, abduction: 60°, adduction: -5°, external rotation: 0°, satisfactory function: Constant-Murley Shoulder Score: 65
4	87	Female	Osteoporosis, cognitive impairment	Glenohumeral osteoarthritis	Cementless RTSA	Fall	Region 3	120 M	-	Immobilization by Sarmiento cast	6 M	Painless, limited elevation, mobility of elevation at 30°, abduction: 10°, adduction: -10°, external rotation: -5°, Constant-Murley Shoulder Score: 25, dissatisfaction
5	87	Female	Osteoporosis cognitive impairment, CKD	Osteonecrosis	Cemented RTSA	Fall	Region 4	180 M	-	Surgery with long LCP	7 M	Painless mobility of elevation at 120°, abduction: 90°, adduction: -10°, external rotation: 5°, Constant-Murley Shoulder Score: 69, satisfactory function
6	78	Female	-	Fracture	Hemi, cement	Fall	Region 3	200 M	-	Surgery with RSTA long stem	6 M	Painless mobility of elevation at°, abduction: 90°, adduction: -20°, external rotation: 5°, Constant-Murley Shoulder Score: 73, satisfactory function

## Discussion

Managing periprosthetic fractures of the shoulder is a challenge, even for experienced surgeons. The treatment goals should focus on fracture healing, maintaining stability of the prosthetic stem, preserving shoulder movement, and restoring overall shoulder function [7,8]. There is limited information available on periprosthetic humeral fractures, and the reported incidence of this type of fracture varies significantly across studies, ranging from 1.2% to 19.4% [1-3].

A study by Singh et al. [9], which included 4,019 prostheses over 33 years, reported an incidence of periprosthetic fractures of 2.6%. Another series by Wagner et al. [10] observed an incidence of 16% in 231 prostheses.

In our series, the incidence of this type of fracture was 6.1%, all occurring postoperatively. However, the incidence is expected to increase in the coming years due to the rising use of shoulder prosthetic implants in both trauma and elective orthopedic surgery.

Several risk factors have been identified, including advanced age, female sex, rheumatoid arthritis, osteoporosis, osteopenia, avascular necrosis of the humeral head, and technical factors such as extensive reaming of the medullary canal, aggressive manipulation, and soft tissue contracture [6].

Many postoperative periprosthetic humeral fractures occur after a simple fall from standing height on the operated limb. They can also occur without apparent trauma due to the bone fragility of the cortices, exacerbated by the increased stresses at the periprosthetic level.

Several classification systems have been reported in the literature, most involving the identification of the anatomical location of the fracture and the evaluation of implant stability.

The fractures in our series are classified according to Campbell's system. This system categorizes fractures based on the location of the fracture line. It assumes that fractures of the proximal humeral metaphysis have different implications for consolidation and prosthetic stability compared to those occurring in the diaphysis. This classification helps guide the therapeutic approach based on radiographic and intraoperative findings [11].

For periprosthetic humeral fractures, conservative treatment may be an option if the alignment is acceptable and there is no prosthetic loosening [12-15]. However, some studies have shown that patients who received nonsurgical treatment were not very satisfied. Kumar et al. [15] observed bone healing in five out of 11 cases, while Boyd et al. [11] had similar results in one out of seven cases.

In our series, conservative treatment was attempted in three patients due to associated comorbidities, patients' refusal of surgical intervention, and limited functional demand. Consolidation was achieved in two out of three patients, with only one patient recovering satisfactory function for performing daily activities.

The generally accepted indications for surgical treatment of periprosthetic humeral fractures are displaced or unstable fractures, along with fractures around a loose humeral component [16-18]. When the patients' overall condition allowed, a CT scan was systematically performed to assess bone quality and plan the surgical intervention. In our series, three patients underwent surgical treatment, which led to fracture healing and satisfactory functional recovery.

The main limitations of our study are the small number of included patients, specific pathological profile, advanced age of our patients, and their limited functional demand, which restricted the planned therapeutic project.

Periprosthetic humeral fractures following shoulder arthroplasty present a significant challenge for surgeons. The incidence of this type of fracture is steadily increasing. Various classification systems exist, taking into account factors such as fracture location, orientation, proximity to the prosthesis, and implant stability. However, these classification systems have limitations in guiding treatment or predicting outcomes. Fortunately, new systems are emerging and offering promising perspectives in this field.

According to current literature, bracing is recommended as the first-line treatment for postoperative periprosthetic fractures with stable components and acceptable alignment. However, failure rates seem to be high in these cases. On the other hand, surgical intervention is recommended if there is prosthetic loosening, non-union, or an inability to maintain stability with bracing.

The development of humeral stem designs, including short stems, stemless components, uncemented components, and implants with reduced canal fill, is expected to reduce the risk of periprosthetic fractures and lead to different fracture patterns. However, further research is necessary to establish the best treatment approaches, particularly in cases of severe bone loss.

## Conclusions

Periprosthetic fractures of the humerus are a rare but increasingly common complication due to the rising number of shoulder prostheses. These fractures pose significant challenges for surgeons, requiring careful management to ensure bone healing and implant stability. Our retrospective study over a four-year period highlighted the complexity of treating these fractures, with patient outcomes varying based on the treatment approach and individual patient factors.

Surgical intervention generally resulted in better functional recovery compared to conservative management, although complications such as radial nerve palsy were noted. All fractures healed within an average of seven months, with surgical patients achieving satisfactory shoulder function. The study underscores the importance of a tailored, multidisciplinary approach to optimize treatment outcomes. Advances in humeral stem design and individualized treatment strategies will be crucial in addressing this growing clinical challenge.
